# Berberine nanoemulsion against nephrotoxicity induced by bisphenol a in rats during pre-puberty stage

**DOI:** 10.1016/j.toxrep.2026.102203

**Published:** 2026-01-08

**Authors:** Shahnaz Rajabi, Atena Mansouri, Tahora Fakhrtaha, Parisa Sadighara, Fariborz Samini, Saeed Samarghandian, Tahereh Farkhondeh

**Affiliations:** a Medical Toxicology Research Center, Mashhad University of Medical Sciences, Mashhad, Iran; b Cellular and Molecular Research Center, Birjand University of Medical Sciences, Birjand, Iran; c Department of pathology, Ghaem Hospital, Mashhad University of Medical Sciences, Mashhad, Iran; d Department of Environmental Health Engineering, School of Public Health, Tehran University of Medical Sciences, Tehran, Iran; e Neuroscience Research Center, Mashhad University of Medical Sciences, Mashhad, Iran; f Department of Medical Physiology, School of Medicine, Neyshabur University of Medical Sciences, Neyshabur, Iran; g Geriatric Health Research Center, Birjand University of Medical Sciences, Birjand, Iran

**Keywords:** Berberine nanoemulsion, Bisphenol, Nephrotoxicity, Rat, Pre-puberty

## Abstract

This study evaluated the effects of berberine nanoemulsion (BNE) against nephrotoxicity induced by bisphenol A (BPA), a plastic chemical, in rats during the pre-puberty stage. Thirty-six male Wistar rats (23-day-old) were randomly allocated to six groups (n = 6): Group 1 (Control). Group 2 (BPA): BPA (200 mg/kg) and saline. Groups 3 (BNE 5) and 4 (BNE 10): BNE (5 and 10 mg/kg,). Groups 5 (BPA + BNE 5) and 6 (BPA + BNE 10): BPA with BNE (5 and 10 mg/kg). After 30 days, kidney samples were obtained for histopathological and biochemical tests. BPA increased serum urea, uric acid, creatinine (Cr), blood urea nitrogen (BUN), and kidney levels of malondialdehyde (MDA), nitric oxide (NO), interleukin-6 (IL-6), interleukin-1β (IL-1β), and tumor necrosis factor-α (TNF-α) versus controls. BPA decreased estimated glomerular filtration rate (eGFR), reduced glutathione (GSH), and superoxide dismutase (SOD) activity. BNE (10 mg/kg) reversed these changes to near-control levels. Histopathology showed lumen obliteration of renal tubules, dilated vessels, glomerular atrophy, and large capsular space in the BPA group. Less inflammation and improved glomerular architecture were seen in the BPA + BNE10 group. In conclusion, BNE (10 mg/kg) significantly alleviated BPA-induced oxidative stress, inflammation and histopathological changes in the kidney of rats during the pre-puberty stage due to its antioxidant and anti-inflammatory effects. This finding confirms the safety and efficacy of BNE in nephrotoxic model during pre-puberty stage. It can be suggested to investigate this agent in clinical study as a possible therapeutic approach.

## Highlights

• Bisphenol A (BPA) impaired kidney function in prepubertal rats.
• BPA increased oxidative stress and reduced antioxidant defenses.
• BPA elevated renal pro-inflammatory cytokines (IL-6, IL-1β, TNF-α).
• Berberine nanoemulsion (BNE, 10 mg/kg) reversed biochemical changes.
• BNE alleviated BPA-induced renal injury via antioxidant and anti-inflammatory effects.

## Graphical Abstract

**Figure:**
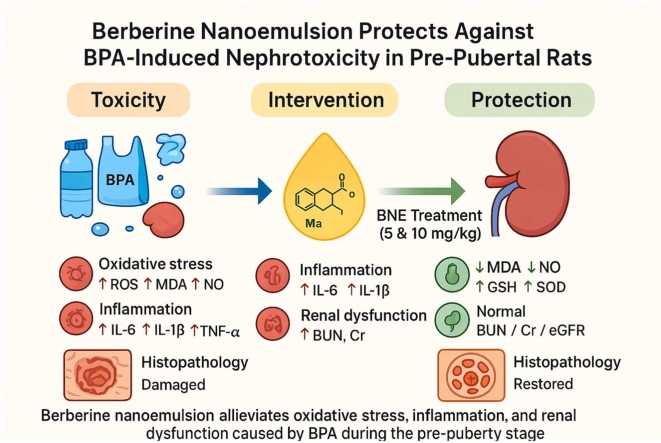

## 1 Introduction

Renal disorders are among the most common diseases that threats human health [1]. The prevalent lack of effective treatment against renal disorders leads to an increased likelihood of mortality [1]. Environmental pollutants are considered as a significant public health concern that can induce acute and chronic kidney diseases [2].

Bisphenol A (BPA) is one of the oldest synthetic compounds with endocrine disrupter activity, that has been used extensively in epoxy resins lining food, beverage containers, plastics and in medical and dental devices including dialyzers [3]. Several studies revealed that the risk of exposure to BPA is increasing in developing country which emphasis the need for assessment of cumulative risk in the different population [3], [4]. These data have increased concerns about the possible implication of BPA in the etiology of various human diseases [4]. In this context, several experimental and epidemiological studies have indicated highly correlated between BPA exposure and concentration with renal dysfunction [5], [6], [7]. In addition, the systematic and meta-analysis studies confirmed that high blood levels of BPA could be a factor in progressing of kidney disease, at least in people with cardiometabolic diseases [4], [8].

Animal studies also indicated that BPA is able to induce direct effect on the kidney mitochondria, causing oxidative stress and inflammation and subsequently, leading to whole organ damage [9], [10]. Important evidences suggest the protective effects of flavonoids against nephrotoxicity induced by BPA, which may be mediated, in part, by its ability to diminish oxidative stress and maintain redox equilibrium within the mitochondria [11], [12].

Berberine is a main isoquinoline type of alkaloid ingredients in several plants including Berberis sp. The high safety of berberine has been confirmed in both human and animals [13]. It has been found that berberine has protective effects against various of diseases including Alzheimer disease, diabetes, cardiotoxicity, nephrotoxicity, etc., [14], [15]. In this context, animal studies reported the protective effect of berberine against nephrotoxicity by decreasing renal malonaldehyde (MDA), nitric oxide (NO) along with nuclear factor-kappa B (NF-KB) renal mRNA expression [16]. In addition, BBH also significantly increased the concentrations of reduced glutathione (GSH), superoxide dismutase (SOD), catalase (CAT), glutathione peroxidase (GPx) and glutathione reductase (GR) in the kidney tissue [16]. However, oral administration of berberine exhibited low bioavailability and poor stability mainly due to its hydrophobic properties resulted from the chemical structure, which contains two methoxy groups and a quaternary ammonium cation [17]. It was found that nanoemulsion formulation significantly improved the absorption and oral bioavailability of berberine. However, the safety and efficacy of berberine nanoemulsion (BNE) has not been fully understood.

Due to high susceptibility of body organs to drug and toxic agents during developmental period, this study was designed to evaluate the effect of BNE against nephrotoxicity induced by BPA in male rat during pre-puberty.

## 2 Materials and methods

### 2.1 Chemicals

Bisphenol A (BPA; CAS No. 80–05–7, purity ≥ 99 %) and berberine hydrochloride (CAS No. 633–65–8, purity ≥ 98 %) were obtained from Jiangsu Co., Ltd., China, and BIOSCIENCE Co., Ltd., China. Blood urea nitrogen (BUN), creatinine (Cr), urea, and uric acid were measured using the Pars Azmoon enzymatic kit (Pars Azmoon Co., Tehran, Iran) on a Roche/Hitachi 912 auto-analyzer. The estimated glomerular filtration rate (eGFR) was calculated based on the measured serum creatinine values using the standard rat-specific formula for creatinine clearance estimation: eGFR (ml/min/1.73 m²) = (0.55 × Body Weight (kg)) / Serum Creatinine (mg/dl). MDA (CAS Number: NS-15023), NO (CAS Number: NS-15043), SOD (CAS Number: NS-15032), and GSH (CAS Number: NS: 15087) kits were obtained from Navand Salamat Co., Iran. Interleukin-1β (IL-1β, CAS Number: ZB-10143C-H9648), interleukin-6 (IL-6, CAS Number: ZB-10090C-H9648), and tumor necrosis factor–alpha (TNF-α, CAS Number: ZB-10082C-H9648) kits were obtained from ZellBio GmbH, Co., Germany.

### 2.2 Nanoemulsion preparation and berberine encapsulation

To synthesize berberine nanoemulsion, an oil-in-water formulation was prepared utilizing Tween 80 as the surfactant and ethanol as the co-surfactant. The nanoemulsion was produced through the ultrasonic method, utilizing a power of 150 watts over a duration of five minutes. Initially, 1000 µl of Tween 80 were introduced into a 50 ml Falcon tube. 500 μl of olive oil was added to the surfactant mixture, followed by the addition of 1000 μl of ethanol to the tube. Subsequently, the appropriate amount of berberine was dissolved in the oil phase. The blend was mixed using a stirrer for up to 30 min. Then the oil phase was slowly added to the water phase while continuously stirring the mixture overnight. The resulting solution was then transferred to an ultrasonic device set at 150 watts of power and sonicated for 5 min to prepare the nanoemulsion. Lastly, the size and morphology of the particles in the solution were analyzed using dynamic light scattering (Nano-ZS, Malvern, UK) and transmission electron microscopy (CM120, Philips) to confirm the formation of the nanoemulsion [18].

### 2.3 Animal study

Thirty-six male Wistar rats, 23 days old, were purchased from the Birjand Experimental Animal Lab Center. We provided standard conditions (22–25ºC temperature, 12 h light/dark cycle, and free access to water and food) for 2 weeks. The study was approved by the ethics committee of the National Institutes for Medical Research Development (NIMAD), Iran (approval number: IR.NIMAD.REC.1403.039). The ARRIVE guideline was employed for reporting experiments involving live animals, promoting ethical research practices.

The study consisted of 6 groups of 6 rats each, treated for 30 consecutive days as follows:

• Group 1 (Control): Rats were orally treated with corn oil alone at 0.3 cc and received 0.3 cc normal saline intraperitoneally.
• Group 2 (BPA alone): Rats were orally treated with BPA at 200 mg/kg and received 0.3 cc normal saline intraperitoneally. In this study, BPA was administered orally at 200 mg/kg body weight for 30 consecutive days [19].
• Group 3 (BNE 5): Rats received BNE intraperitoneally at 5 mg/kg [20].
• Group 4 (BNE 10): Rats received BNE intraperitoneally at 10 mg/kg [20].
• Group 5 (BPA + BNE 5): Rats were co-treated with BPA and BNE at 5 mg/kg.
• Group 6 (BPA + BNE 10): Rats were co-treated with BPA and BNE at 10 mg/kg.

After 30 days, rats were anesthetized with ketamine (50 mg/kg) and xylazine (10 mg/kg), and kidney samples were obtained for histopathological and biochemical tests.

The experimental design (six groups, including appropriate controls, BNE-alone, BPA-alone, and combined BPA+BNE treatment at two different doses) was structured to (1) establish a clear baseline of BPA-induced nephrotoxicity, (2) assess the independent effects of BNE, and (3) evaluate dose-dependent protective effects of BNE. This approach allowed comprehensive assessment of both toxic and protective outcomes, supported by adequate replication (n = 6 per group) and consistent with ethical animal use standards.

### 2.4 Biochemical assays

The urea, uric acid, BUN, and Cr concentrations in the serum samples were measured by auto-analyzer using the Pars Azmoon kit. eGFR was calculated by formula for rodent studies as follow:

eGFR (ml/min/1.73 m²) = (0.55 × Body Weight (kg)) / Serum Creatinine (mg/dl) [21].

### 2.5 Oxidative stress indices measurements

Homogenized kidney samples (100 mg) were mixed with 500 µL of phosphate buffer to measure the concentrations of GSH, SOD, MDA, and NO.

### 2.6 GSH assay

The concentration of GSH in the kidney tissue was determined using Ellman’s assay according to the protocol of commercial kit which was produced by Navand Salamat Co. DTNB (5,5-dithio-bis-(2-nitrobenzoic acid) also known as Ellman's reagent was used for measuring free sulfhydryl groups. First, Glutathione disulfide (GSSG) is reduced to GSH by glutathione reductase (GR) enzyme. For this purpose, DTNB reagent and GR enzyme were added to the samples and incubated for 10 min in the dark at room temperature. GSH react with DTNB to produce yellow TNB and measured spectrophotometrically at 412 nm.

### 2.7 SOD assay

The measurement of SOD activity was performed by inhibition of the pyrogallol autoxidation reaction according to the protocol of commercial kit which was produced by Navand Salamat Co. The inhibition of pyrogallol is grounded in the presence of SOD in cells that reduces the superoxide radicals (O2•−) into O2 and H2O2. Summary, pyrogallol and sodium carbonate (alkaline buffer) were added to samples and mixed. In alkaline solutions, pyrogallol can react with dioxygen and produce purpurogallin, that changes to the dianion of purpurogalloquinone. This product can react with O2•−. Immediately the absorbance of samples was measured spectrophotometrically at 405 nm.

### 2.8 MDA assay

The MDA concentration was measured using the thiobarbituric acid reactive substances (TBARS) method according to the protocol of commercial kit which was produced by Navand Salamat Co. Briefly, the samples was mixed with TCA and TBA reagent. The mixture was incubated in boiling water bath at 95 °C for 45 min. The samples were quickly cooled on ice during 10 min. Then, the samples were centrifuged at 3000 rpm for 15 min. At high temperature, TBA was reacted with MDA and produced the pink complex. The absorbance was measured spectrophotometrically at 550 nm.

### 2.9 NO assay

The NO concentration was measured using Griess reaction according to the protocol of commercial kit which was produced by Navand Salamat Co. Briefly, Griess reagent A (sulfanilamide) was added to samples. Then the samples were incubated for 10 min in room temperature. Griess reagent B (N-1-naphtylethelenddiamine) was added to the samples and incubated for 10 min in room temperature. The absorbance was measured spectrophotometrically at 570 nm.

### 2.10 Inflammatory indices measurement

The IL-1β, IL-6, and TNF-α concentrations in the rat kidney were measured using the sandwich ELISA technology based on the manufacturer’s protocols (ZellBio GmbH, Co.).

#### 2.10.1 Histopathological assay

After sampling, the kidney tissue of the rats was preserved in 10 % formalin. Then, the tissue samples were stored in ethanol and subsequently cleared with xylene. Finally, the specimens were embedded in liquid warm paraffin and cut into 4–5 μm sections. Hematoxylin and eosin stain (H&E) was used for histological staining.

#### 2.10.2 Statistical analysis

Statistical analyses were performed using InStat 3.0 software. Data were shown as mean ± standard deviation (SD). Using the Shapiro–Wilk test, the normality of quantitative variables was examined. If normality was established, analysis of variance (ANOVA) followed by the Tukey test was used to compare the data. A P-value < 0.05 was considered statistically significant.

## 3 Results

### 3.1 Characterization of nanoemulsion

**Fig. 1:**
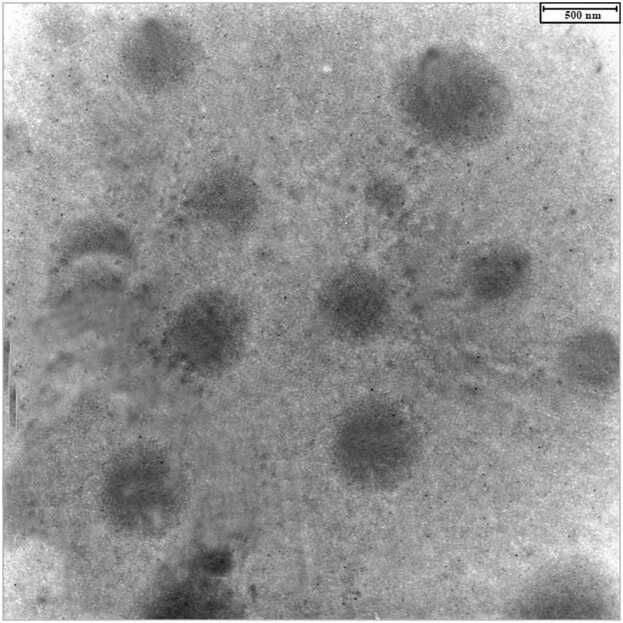
The TEM image of the optimized nanoemulsion formulation at 500 nm scale.

**Fig. 2:**
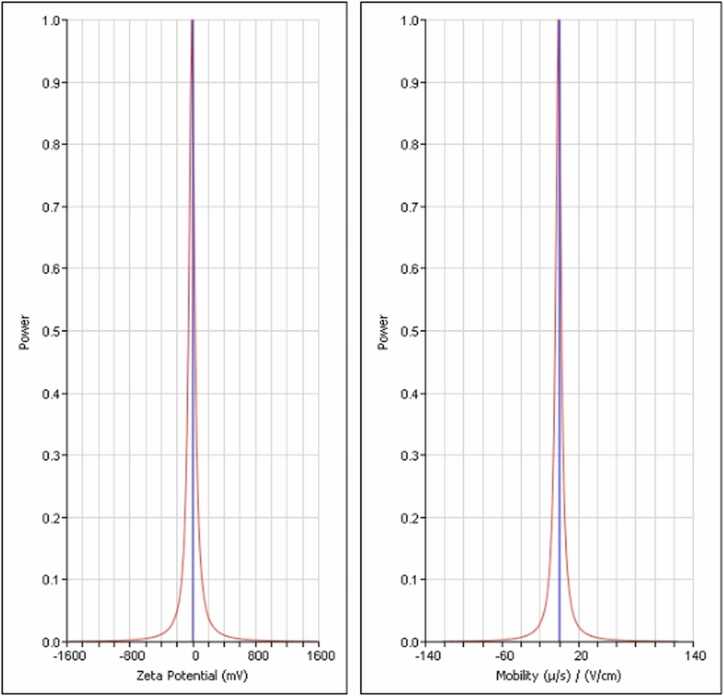
DLS findings. Zeta potential indicates the surface charge of the nanoparticles.

The positive TEM image of the optimized NE revealed spherical droplets devoid of aggregation, consistent with the results obtained from dynamic laser scattering (DLS) analysis (Fig. 1). DLS studies indicated a size of 119.92 nm, accompanied by a polydispersity index and zeta potential of 0.33 mV (Table 1, Fig. 2).

**Table 1:** DLS findings.

**Sample ID**	**Z-Average (nm)**	**PDI**	**Mean Diameter by Number (nm)**	**Zeta Potential (mV)**
NBE-	115.85 ± 1.90	0.117 ± 0.002	95.21 ± 10.77	-13.11

Data are presented as mean ± standard deviation (n = 3). Z-Average represents the hydrodynamic diameter measured by DLS, PDI indicates the polydispersity index, and Mean Diameter by Number represents the particle size distribution based on particle count. Zeta potential indicates the surface charge of the nanoparticles.

### 3.2 Findings of renal function indexes

Effect of BPA on renal function of rats in exposed group indicated the significant increase in the serum concentrations of urea (p < 0.001), uric acid (p < 0.01), Cr (p < 0.01) and BUN (p < 0.001) as in comparison to the control group. In addition, the eGFR significantly decreased in the BPA group in comparison to the control group (p < 0.001). Treatment with BNE (10 mg/kg) had no effect on the serum concentrations of urea, uric acid, Cr, BUN and eGFR in the BPA-exposed group in comparison to the non-treated BPA group.

Supplementation of BNE (10 mg/kg) reversed BUN, Cr, urea, uric acid and eGFR induced by BPA to the levels that were comparable to the control group (p < 0.05 for all parameter) (Table 2).

**Table 2:** Comparison of concentrations of kidney functional indices in the serum of experimental groups.

Groups Parameters	C	BPA	BNE 5	BNE 10	BNE 5 +BPA	BNE 10 +BPA
Urea (mg/dl)	27.50 ± 5.12	42.08 ± 6.35 ***	35.66 ± 7.09	36.08 ± 2.73	37.01 ± 4.41 *	33.66 ± 3.02 +
Cr (mg/dl)	0.54 ± 0.17	0.76 ± 0.08 **	0.58 ± 0.04	0.56 ± 0.07	0.75 ± 0.06 *	0.60 ± 0.05 +
Uric acid (mg/dl)	0.96 ± 0.39	2.11 ± 0.86 **	1.34 ± 0.53	1.29 ± 0.50	1.85 ± 0.30 **	1.25 ± 0.13
BUN (mg/dl)	11.70 ± 2.13	19.02 ± 1.56 ***	14.82 ± 2.24	15.11 ± 2.06	18.55 ± 3.98 ***	15.75 ± 1.89 +
eGFR (ml/min/1.73 m²)	257.25 ± 69.07	164.08 ± 11.18 ***	201.98 ± 23.58	197.66 ± 29.27	179.08 ± 15.57**	221.05 ± 20.80 +

Data are shown as means ± SD for each group (n = 6).

C: Control, BPA: Bisphenol a, BNE 5: Berberine hydrochloride nanoemulsion 5 mg/kg, BNE 10: Berberine hydrochloride nanoemulsion 10 mg/kg, BNE 5 +BPA: Berberine hydrochloride nanoemulsion 5 mg/kg + Bisphenol a, BNE 10 +BPA: Berberine hydrochloride nanoemulsion 10 mg/kg + Bisphenol a.

A significant difference between the data of the C group vs. other groups: *; p < 0.05, **; p < 0.01, ***; p < 0.001.

A significant difference between the data of the BPA group vs. other treatment groups: + ; p < 0.05.

### 3.3 Findings of oxidative stress indexes

As shown in Table 3, there were a significant increase in the concentrations MDA (p < 0.001) and NO (p < 0.001) of BPA exposed group in comparison to the control group. Pretreatment with CA significantly improved MDA concentration (p < 0.05). In addition, the activity of SOD (p < 0.001) and GSH (p < 0.01) concentration decreased in BPA-exposed groups compared to control group. Treatment with BNE (10 mg/kg) significantly decreased the concentrations of MDA and NO, and also increased GSH concentration and the SOD activity in the kidney of the BPA-exposed group in comparison to the non-treated BPA group (p < 0.05 for all parameter).

**Table 3:** Comparison of concentrations of oxidative stress indexes in the kidney of experimental groups.

Groups Parameters	C	BPA	BNE 5	BNE 10	BNE 5 +BPA	BNE 10 +BPA
GSH (µM)	24.62 ± 2.47	19.19 ± 1.44 **	22.83 ± 2.20	23.07 ± 2.15	19.47 ± 2.90 **	21.66 ± 2.58 + ,&
SOD (U/mg tissue)	18.33 ± 1.08	14.43 ± 0.98 ***	19.62 ± 1.15	19.29 ± 1.24	14.62 ± 1.87 ***	16.37 ± 0.66 +
MDA (nM/mg tissue)	32.29 ± 3.65	46.88 ± 1.85 ***	33.23 ± 2.78	34.86 ± 4.93	43.98 ± 2.69 ***	42.10 ± 2.05 +
NO (µM)	93.05 ± 4.47	116.60 ± 8.80***	96.80 ± 4.65	89.33 ± 6.69	113.89 ± 8.35 ***	105.29 ± 5.89 +

Data are shown as means ± SD for each group (n = 6).

C: Control, BPA: Bisphenol a, BNE 5: Berberine hydrochloride nanoemulsion 5 mg/kg, BNE 10: Berberine hydrochloride nanoemulsion 10 mg/kg, BNE 5 +BPA: Berberine hydrochloride nanoemulsion 5 mg/kg + Bisphenol a, BNE 10 +BPA: Berberine hydrochloride nanoemulsion 10 mg/kg + Bisphenol a.

A significant difference between the data of the C group vs. other groups: **; p < 0.01, ***; p < 0.001.

A significant difference between the data of the BPA group vs. other treatment groups: + ; p < 0.05.

A significant difference between the data of the BNE 5 +BPA group vs. BNE 10 +BPA group: &; p < 0.05.

### 3.4 Findings of inflammatory indexes

There was a significant increase in the concentrations of IL-6 (p < 0.001), IL-1β (p < 0.05), and TNF-α (p < 0.001) in the kidney tissue of the BPA group in comparison to the control group.

The significant decrease in the L-6 (p < 0.05), IL-1β (p < 0.05), and TNF-α (p < 0.05) was found between BPA+BNE 10 group and BPA group. It was also observed the remarkable difference between BPA+BNE 10 group versus BPA+BNE 5 group (P < 0.05) (Table 4).

**Table 4:** Comparison of concentrations of inflammatory indexes in the kidney of experimental groups.

Groups Parameters	C	BPA	BNE 5	BNE 10	BNE 5 +BPA	BNE 10 +BPA
IL-6 (pg/ml)	21.43 ± 0.55	24.71 ± 1.70 ***	20.78 ± 1.11	21.39 ± 1.28	23.37 ± 0.72	23.11 ± 1.40 +
IL-1β(pg/ml)	45.94 ± 1.22	49.61 ± 1.67 *	45.21 ± 2.22	44.10 ± 1.85	47.99 ± 1.72	47.20 ± 1.94 +
TNF-α (pg/ml)	15.86 ± 0.86	20.72 ± 1.39 ***	15.69 ± 1.07	15.74 ± 1.78	20.40 ± 1.34 ***	19.04 ± 2.53 *, + , &

Data are shown as means ± SD for each group (n = 6).

C: Control, BPA: Bisphenol a, BNE 5: Berberine hydrochloride nanoemulsion 5 mg/kg, BNE 10: Berberine hydrochloride nanoemulsion 10 mg/kg, BNE 5 +BPA: Berberine hydrochloride nanoemulsion 5 mg/kg + Bisphenol a, BNE 10 +BPA: Berberine hydrochloride nanoemulsion 10 mg/kg + Bisphenol a.

A significant difference between the data of the C group vs. other groups: *; p < 0.05, ***; p < 0.001.

A significant difference between the data of the BPA group vs. other treatment groups: + ; p < 0.05.

A significant difference between the data of the BNE 5 +BPA group vs. BNE 10 +BPA group: &; p < 0.05.

### 3.5 Findings of histopathological findings

**Fig. 3:**
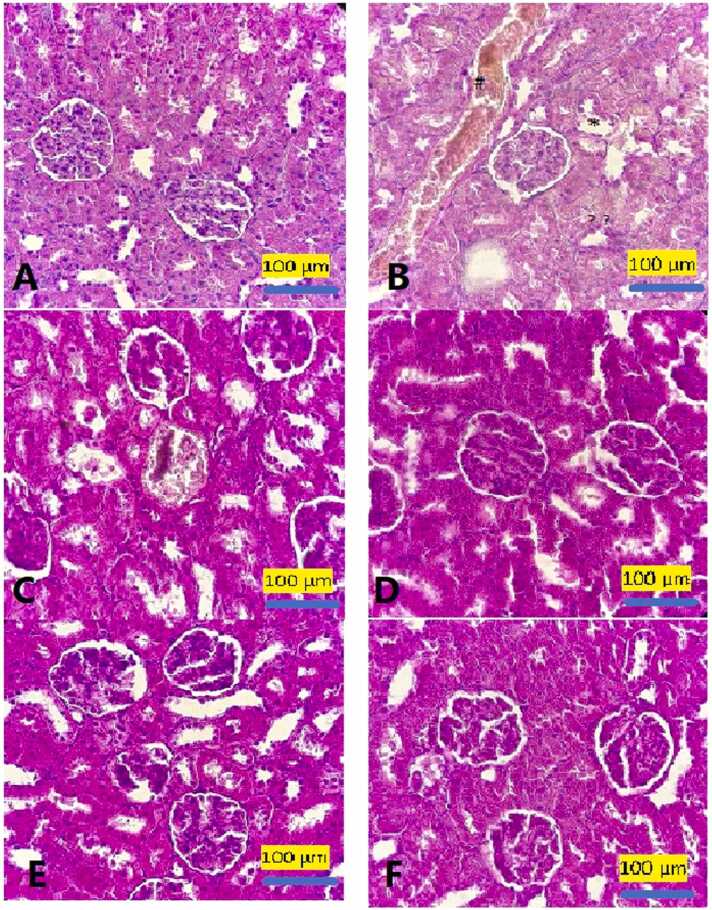
Photomicrographs of hematoxylin and eosin (H&E)- stained sections of the kidney of control rats indicating (A) normal histological structure of the glomeral tissue. B: BPA group with oblitation of the lumen of some renal tubules(*), dilated blood vessel (#), atrophy of the glomerulus and large capsular space (*), vacuolation and necrosis (↑↑) of most tubular epithelial cells. C.BPA+ BNE 5: BPA group treated by BNE shows hemmorrage(H). D.BPA+ BHB10 showed less inflamation and improved glumeral architcture. E: BNE 5 and F:BNE 10 groups showed the normal histological structure of the glomeral tissue.

In the control group, glomerular tissue had a normal structure (Fig. 3A). Obliteration of the lumen of some renal tubules, dilated blood vessel, atrophy of the glomerulus and large capsular space was found in BPA group (Fig. 3B). Hemorrhage was found in BPA group treated with BNE 5 (Fig. 3C). BPA+ BNE10 showed less inflammation and improved glomera architecture (Fig. 3D). Normal structure of glomerular tissue was found in BNE 5 and BNE10 groups (Fig. 3E and F). Table 5 indicated the pathological scoring of the kidney tissues of all experimental groups.

**Table 5:** Histopathological scoring of the kidney tissues.

Groups Pathological scoring	C	BPA	BNE 5	BNE 10	BNE 5 +BPA	BNE 10 +BPA
Dilated blood vessel	1	1.3	1	1	1	1
Large capsular space	1	2	1	1	1.8	1.6
Tubular epithelial necrosis	1	1.5	1	1	1	1
Lymphocytic infiltration	1	1	1	1	1	1
Obliteration of the lumen of some renal tubules	1	1.6	1	1	1	1
Hemorrhage	1	1	1	1	1.5	1.3

C: Control, BPA: Bisphenol a, BNE 5: Berberine hydrochloride nanoemulsion 5 mg/kg, BNE 10: Berberine hydrochloride nanoemulsion 10 mg/kg, BNE 5 +BPA: Berberine hydrochloride nanoemulsion 5 mg/kg + Bisphenol a, BNE 10 +BPA: Berberine hydrochloride nanoemulsion 10 mg/kg + Bisphenol a.

Score 1: No observed the histopathological changes.

Score 2: Observed the histopathological changes.

## 4 Discussion

Human and animal in pre-puberty period are at increased risk for decrease in kidney function [22]. It was found that the decline of eGFR is 10 times faster in children with kidney disease in pre-puberty period than after puberty [22]. The kidney is very susceptible to nephrotoxic agent during pre-puberty period [22]. The limited epidemiological studies indicated the association between BPA exposure and children with CKD and suggested to perform more evaluation of the effects of this exposure in high risk population [23], [24]. Regarding to the challenges in the assessment of human exposure and tissue sampling, it is important to perform to evaluate BPA effect in animals during pre-puberty period for extrapolating the results to humans. Although several animal studies have indicated the nephrotoxic effects of BPA but there is no finding about the BPA effects on the kidney of animals during pre-puberty.

The outcomes of our study demonstrated that BPA exposure for 4 weeks caused azotemia, as shown by increases in urea, uric acid, Cr and BUN concentration in rat during pre-puberty. These data indicated that BPA has a negative effect on the kidney and resulted in renal dysfunction.

The ability of kidney to excrete urine is impaired following a defect in tubular function and/or glomerular filtration [25]. Interestingly in our study, a marked decrease in eGFR was also found in animals exposed to BPA. It is proposed that the renal dysfunction induced by BPA might be associated with toxic effect of this agent on the glomerulus and tubular epithelial cells. This suggestion was confirmed by our histopathological finding indicating glomerular atrophy and large capsular space, vacuolation and necrosis of most tubular epithelial cells in BPA exposed animals.

This result was consistent with the previous studies indicating BPA-induced glomerular and tubular injury in adult animal models as well as a significant diminished in creatinine clearance and an increased in urine protein excretion [26], [27].

Although the main toxicity mechanism of BPA may be associated with its effects on hormone receptors; however, the role of oxidative stress in BPA-induced nephrotoxicity is significant [28]. Oxidative stress is induced following disturbances in the balance between the antioxidant system and reactive oxygen species (ROS) production [28]. This imbalance in the pro-oxidant-antioxidant may cause oxidative damage of lipids as evidenced by an elevation lipid peroxidation marker (MDA) and also NO concertation with a decrease in cellular kidney antioxidant content and antioxidant enzymes activity [28]. The biochemical findings of this study indicated disruption of oxidant-antioxidant balance (decreased SOD activity and GSH level and also increased MDA and NO levels). Similar to our findings, Kobroob et al., Chen et al., and Nagarajan et al. indicated the induction of lipid peroxidation and NO with reduction of GSH and antioxidant enzyme in the kidney of adult male rats exposed to BPA [27], [29], [30].

Overproduction of ROS can also induce the release pro-inflammatory cytokines and chemokines. Thus, oxidative stress is responsible for in the initiation and progression of the inflammatory signaling. In addition, increased ROS and inflammation can exacerbate mitochondrial dysfunction and leading to cell death [31].

In this context, we found an increase in TNF-α, IL-6 and IL-1β concentrations in the kidney of rats during pre-puberty stage exposed to BPA. This data is similar to earlier studies indicating an increase in pro-inflammatory cytokines following BPA exposure [32], [33]. It may be related to ROS overproduction that is triggered renal inflammation or direct induction of the pro-inflammatory cytokines by BPA [32]. The observed inflammation in histopathological evaluation was further confirmation for induction of inflammation by BPA in this study.

Recent investigations indicated that berberine was able to protect against nephrotoxicity [34], [35]. However, its weak aqueous solubility, low absorption and rapid metabolism limited its application. Synthesis of berberine nanoparticle formulations improved its bioavailability [36].

The present study indicated that co-administration of BNE at dose 10 mg/kg was able to diminish BPA-induced nephrotoxicity, as evidenced by the improvements in azotemia, eGFR and renal histopathological changes. In this study, the protective effect of BNE was found in related to the restorations of some oxidative indexes (MDA, NO, GSH and SOD) in the kidney tissues of BPA-exposed rats, proposing that renoprotective effect of BNE is due to its antioxidant activity. This confirmed the previous studies indicating the free radical scavenging and antioxidant activity of berberine nanoparticle [36]. It has been also indicated that BNE at dose 10 mg/kg decreased the concentrations of pro-inflammatory cytokines including TNF-α, IL-6 and IL-1β.

The BNE (10 mg/kg) reversed the increased TNF-α, IL-6 and IL-1β concentrations to normal levels suggested TNF-α, IL-6 and IL-1β has anti-inflammatory activity in the kidney of male rats during pre-puberty stage. Similar to our finding, previous studies indicated that berberine and its nano-formulations were able to diminish the increased levels of TNF-α, IL-6 and IL-1β in several tissues [37]. In addition, Xie et al. fond that administration of berberine and berberine nanoparticles (2 and 4 mg/kg) 6 h and 24 h after the construction of ischemia-reperfusion protected kidney function as evidenced by normal serum urea nitrogen and Cr concentration and histology against damage induced by ischemia in rats via inhibiting oxidative stress and apoptosis of renal cells [38]. Wu et al. indicated the biofabricate berberine coated nano‑silver (75 mg/kg, orally) ameliorated renal injury induced by acetaminophen in diabetic rats by decreasing lipid peroxidation and increasing antioxidants in the kidney. They also found that biofabricate berberine coated nano‑silver could modulate nuclear transcription factor (NF-kB) in kidney tissue [39].

Altogether, these findings confirms the efficacy of BNE against the inflammatory kidney diseases due to the anti-inflammatory effect in this tissue.

Our histopathological assessment confirmed the biochemical findings related to the protective effect of BNE at dose 10 mg/kg against BPA. BNE at dose 10 mg/kg decreased inflammation, necrosis, congestion and atrophy in the kidney tissues.

This study has some limitations. First, although we found a dose dependent effect between the GSH and TNF-α levels in the kidney of BPA-exposed rats treated with BNE (10 mg/kg) versus BPA-exposed rats treated with BNE (10 mg/kg), but it is not enough to conclude the dosage-dependent effects of BNE due to included two doses.

## 5 Conclusion

This study indicated that BPA (200 mg/kg) for 4 weeks was able to cause renal dysfunction via inducing glomerular and tubular damage in the kidney tissue of rats during pre-puberty stage. The significant increase in the levels of NO, MDA, TNF-α, IL-6 and IL-1β with marked reduction in the GSH level and SOD activity in the kidney of rats showed the role of oxidative stress and inflammation in the nephrotoxicity induced by BPA. The reverse of renal function, oxidative stress and inflammatory indexes to normal levels in the kidney of rats treated with BNE (10 mg/kg) indicated the effective anti-inflammatory and antioxidant activity of this agent.

According to our knowledge, this is the first study on the safety and efficacy of BNE in renal dysfunction model in pre-puberty stage which confirmed by normal levels of serum makers of kidney function and histopathology of tissue samples of BNE 5 and 10 groups. However, our study has several limitations including the restricted doses, lowest number of animals in each groups and lack of assessment of upstream signaling pathways involved in oxidative stress and inflammation. Therefore, this study suggests to design more experimental studies to evaluate the detailed of molecular mechanisms involved in the protective effects of BNE. we evaluated the safety and efficacy of BNE in sub-acute study that is better to perform chronic study to find more about the safety of BNE.

And also several clinical trials are needed for evaluating BNE effect in children with kidney diseases.

## CRediT authorship contribution statement

**Parisa Sadighara:** Writing – original draft, Investigation. **Tahora Fakhrtaha:** Methodology, Investigation. **Atena Mansouri:** Methodology, Investigation. **Shahnaz Rajabi:** Writing – original draft, Investigation. **Tahereh Farkhondeh:** Writing – review & editing, Supervision, Methodology, Funding acquisition. **Saeed Samarghandian:** Writing – review & editing, Supervision, Methodology, Conceptualization. **Fariborz Samini:** Writing – review & editing, Methodology.

## Ethics approval and consent to participate

This study was approved by the ethics committee of the National Institutes for Medical Research Development (NIMAD), Iran, Approval number: IR.NIMAD.REC.1403.039. The ARRIVE guidelines were employed for reporting experiments involving live animals, promoting ethical research practices.

## Consent for publication

Not applicable.

## Funding

The research presented in this publication received support from the Elite Researcher Grant, granted under the award number [4010234], from the National Institutes for Medical Research Development (NIMAD) in Tehran, Iran.

## Declaration of Competing Interest

The authors declare that they have no known competing financial interests or personal relationships that could have appeared to influence the work reported in this paper.

## Data Availability

Data will be made available on request.
